# alpha-Sarcin catalytic activity is not required for cytotoxicity

**DOI:** 10.1186/1471-2091-10-9

**Published:** 2009-04-03

**Authors:** Spencer C Alford, Joel D Pearson, Amanda Carette, Robert J Ingham, Perry L Howard

**Affiliations:** 1 Centre for Biomedical Research, University of Victoria, PO Box 3020 Station CSC Victoria, British Columbia, V8W 3N5, Canada; 2 Department of Biochemistry and Microbiology, University of Victoria, PO Box 3055 Station CSC Victoria, British Columbia, V8W 3P6, Canada; 3 Department of Biology, University of Victoria PO Box 3020 Station CSC Victoria, British Columbia, V8W 3N5, Canada; 4 Department of Medical Microbiology and Immunology, University of Alberta, Edmonton, Alberta, T6G 2H7, Canada

## Abstract

**Background:**

α-Sarcin is a protein toxin produced by *Aspergillus giganteus*. It belongs to a family of cytotoxic ribonucleases that inactivate the ribosome and inhibit protein synthesis. α-Sarcin cleaves a single phosphodiester bond within the RNA backbone of the large ribosomal subunit, which makes the ribosome unrecognizable to elongation factors and, in turn, blocks protein synthesis. Although it is widely held that the protein synthesis inhibition caused by the toxin leads to cell death, it has not been directly shown that catalytically inactive mutants of α-sarcin are non-toxic when expressed directly within the cytoplasm of cells. This is important since recent studies have cast doubt on whether protein synthesis inhibition is sufficient to initiate apoptosis.

**Results:**

In this report, we assay α-sarcin cytotoxicity and ability to inhibit protein synthesis by direct cytoplasmic expression. We show that mutations in α-sarcin, which impair α-sarcin's ability to inhibit protein synthesis, do not affect its cytotoxicity. The mutants are unable to activate JNK, confirming that the sarcin-ricin loop remains intact and that the α-sarcin mutants are catalytically inactive. In addition, both mutant and wildtype variants of α-sarcin localize to the nucleus and cytoplasm, where they co-localize with ribosomal marker RPS6.

**Conclusion:**

We conclude that although protein synthesis inhibition likely contributes to cell death, it is not required. Thus, our results suggest that α-sarcin can promote cell death through a previously unappreciated mechanism that is independent of rRNA cleavage and JNK activation.

## Background

α-Sarcin is a small fungal ribonuclease secreted by *Aspergillus giganteus*. It functions as a ribonuclease by catalytically cleaving a single phosphodiester bond in a well-defined RNA motif (the sarcin-ricin loop) within the rRNA scaffold of the large ribosomal subunit. This makes the ribosome unrecognizable to elongation factors and in turn, blocks protein synthesis [1]. In addition to its ability to inactivate the ribosome, α-sarcin selectively interacts with acidic phospholipids, and causes membrane fusion, as well as aggregation of artificial liposomes [2-4]. α-Sarcin's lipid binding properties allow for its uptake into the cell via acidic endosomes [4]. The mechanism of endosomal escape has not been characterized, but Golgi trafficking may be involved [5,6].

α-Sarcin induces cell death through apoptosis but the mechanism is not completely understood [5]. It has been reported that the toxicity of α-sarcin is due to catalytic inactivation of the ribosome, and subsequent inhibition of protein synthesis [5]. Studies in which purified recombinant α-sarcin is added to the exterior of cells have supported this conclusion and suggested that cleavage of the sarcin-ricin loop within the ribosome is a prerequisite for apoptosis in a manner that is JNK dependent [5,6].

Several mutagenesis studies have been conducted to identify the key residues critical for α-sarcin function. The majority of these studies have been in vitro determinations of α-sarcin enzymatic activity against ribosome containing fractions or dinucleotide substrates. In vivo analysis of α-sarcin activity has been limited to application of purified α-sarcin to the exterior of the cells [4]. From these studies, and analogy to α-sarcin relative RNAse T1, the catalytic mechanism of α-sarcin ribonuclease activity has been elucidated. Cleavage within the rRNA proceeds through a 2',3'-cyclic intermediate that is catalyzed by hydrogen transfer from Histidine-137 to Glutamate-96 [7]. This results in transphosphorylation in which the phosphate group is shared between the 2' and 3' positions on the ribose ring [7]. The hydrolysis of this cyclic derivative is catalyzed by these same two residues, however, in this case, Glutamate-96 acts as a general acid, and Histidine-137 acts as the general base [7]. Arginine-121 has also been shown to be important for catalysis as mutations in this residue render α-sarcin unable to cleave ribosomal rRNA, although it retains significant activity against dinucleotides [8]. Arginine-121 of α-sarcin is thought to help position the substrate for catalysis [8].

In this report, we assayed α-sarcin cytotoxicity and its ability to inhibit protein synthesis by direct cytoplasmic expression of the toxin in mammalian cells. We show that mutations in α-sarcin, which impair α-sarcin's ability to block translation of two independent reporters, retain the ability to kill cells. We conclude that protein synthesis inhibition is not necessary for α-sarcin to induce cell death. Instead, our results indicate that α-sarcin can initiate cell death independent of rRNA cleavage and JNK activation.

## Results and discussion

**Figure 1:**
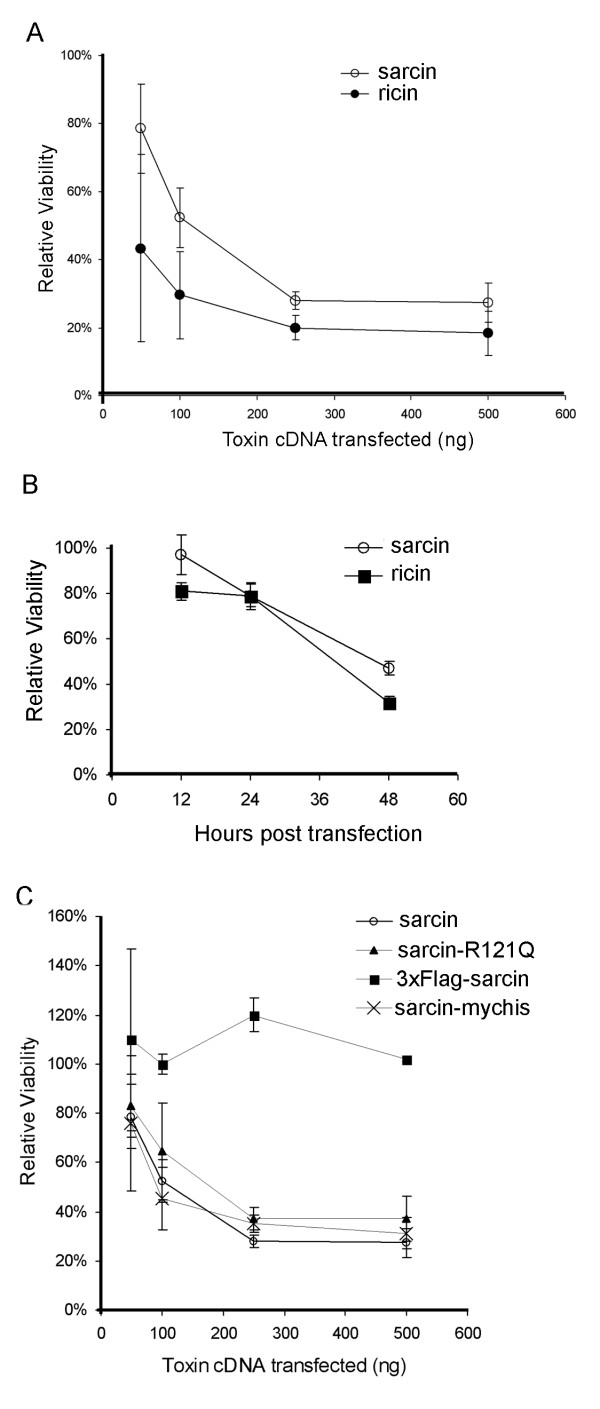
**A) α-Sarcin and ricin induced cytotoxicity in HeLa cells is dose-dependent**. HeLa cells were transfected with increasing amounts of pcDNA3-α-sarcin or pcDNA3-ricin, and assayed for viability, relative to a pcDNA3 control, at 48 hours post-transfection. B) HeLa cells were transfected with α-sarcin or ricin cDNA and assayed for viability, relative to a pcDNA3 control transfection, over a 48 hour time course. C) HeLa cells were transfected with increasing amounts of α-sarcin, α-sarcin-R121Q, an N-terminal 3 × Flag-tagged-sarcin, or a C-terminal mychis-tagged-sarcin and assayed for viability, relative to a pcDNA3 control, at 48 hours post-transfection. Data represents the compiled results of at least 3 independent experiments, each performed in triplicate, +/- standard deviation.

We established a mammalian cell expression system to directly assay the ability of α-sarcin to inhibit translation and induce cell death by expressing the ribotoxin directly in the cytoplasm of HeLa cells. Our objective was to determine the relative cytotoxicity of α-sarcin mutants independent of effects due to toxin entry and trafficking. The α-sarcin cDNA, and for comparison purposes, the ricin toxin A chain cDNA, were cloned into the mammalian expression vector, pcDNA3.1, where gene expression is under control of the constitutive cytomegalovirus (CMV) promoter. Ricin is a glycosidase which depurinates a highly conserved adenine within the sarcin-ricin loop, adjacent to the α-sarcin cleavage site [6]. HeLa cells have been reported to be resistant to α-sarcin activity [9]. This is presumably due to an inability of the toxin to enter HeLa cells. To determine the sensitivity of HeLa cells to direct ribotoxin expression, a dose response analysis was carried out by transfecting cells with increasing amounts of α-sarcin or ricin cDNA. Although both α-sarcin and ricin transfected cells demonstrated a dose-dependent decrease in viability at 48 hours, ricin was more effective at low doses (50 ng) than α-sarcin (Figure 1a). This was consistent with reported catalytic activities of α-sarcin and ricin, with ricin demonstrating approximately 100-fold greater catalytic activity than α-sarcin [10,11]. At concentrations greater than 250 ng, no further reduction in viability was obtained and approximately 20% of the cells remained viable. This was in keeping with our transfection efficiency in HeLa cells (70–85%), which was determined by transfection of pcDNA-GFP reporter plasmid and counting the percentage of fluorescent cells (data not shown). To examine the time course of toxin induced cell death, HeLa cells were transfected with 250 ng of α-sarcin or ricin cDNA, and cell viability was measured over a 48-hour period. Exogenous expression of both α-sarcin and ricin in HeLa cells resulted in decreased viability within 48 hours (Figure 1b). Both α-sarcin and ricin induced cell death at similar rates, with ricin exhibiting slightly enhanced cytotoxicity. Collectively, these results demonstrate that direct cytoplasmic expression of α-sarcin and ricin in mammalian cells is cytotoxic. We next examined whether α-sarcin catalytic activity was required for the cytotoxicity observed during direct cytoplasmic expression.

Based on *in vitro *biochemical assays and cell culture experiments performed with purified α-sarcin, it has been reported that α-sarcin's toxicity is due to its catalytic ability to inactivate the ribosome and inhibit protein synthesis [5]. We sought to determine whether catalytic activity of α-sarcin is required for cytotoxicity when the toxin is directly expressed in the cytoplasm of cells. We initially characterized a catalytic mutant of α-sarcin, α-sarcin-R121Q [8]. Arginine 121 is located near the catalytic site and although it is not directly involved in catalysis, it is thought to help position the substrate in the correct orientation for cleavage [8]. The α-sarcin-R121Q mutant is catalytically attenuated based on *in vitro *biochemical assays [8]. Despite its reduced catalytic ability *in vitro*, expression of α-sarcin-R121Q in HeLa cells resulted in a dose-dependent decrease in viability that was only slightly reduced from that observed for wild-type α-sarcin (Figure 1c).

**Figure 2:**
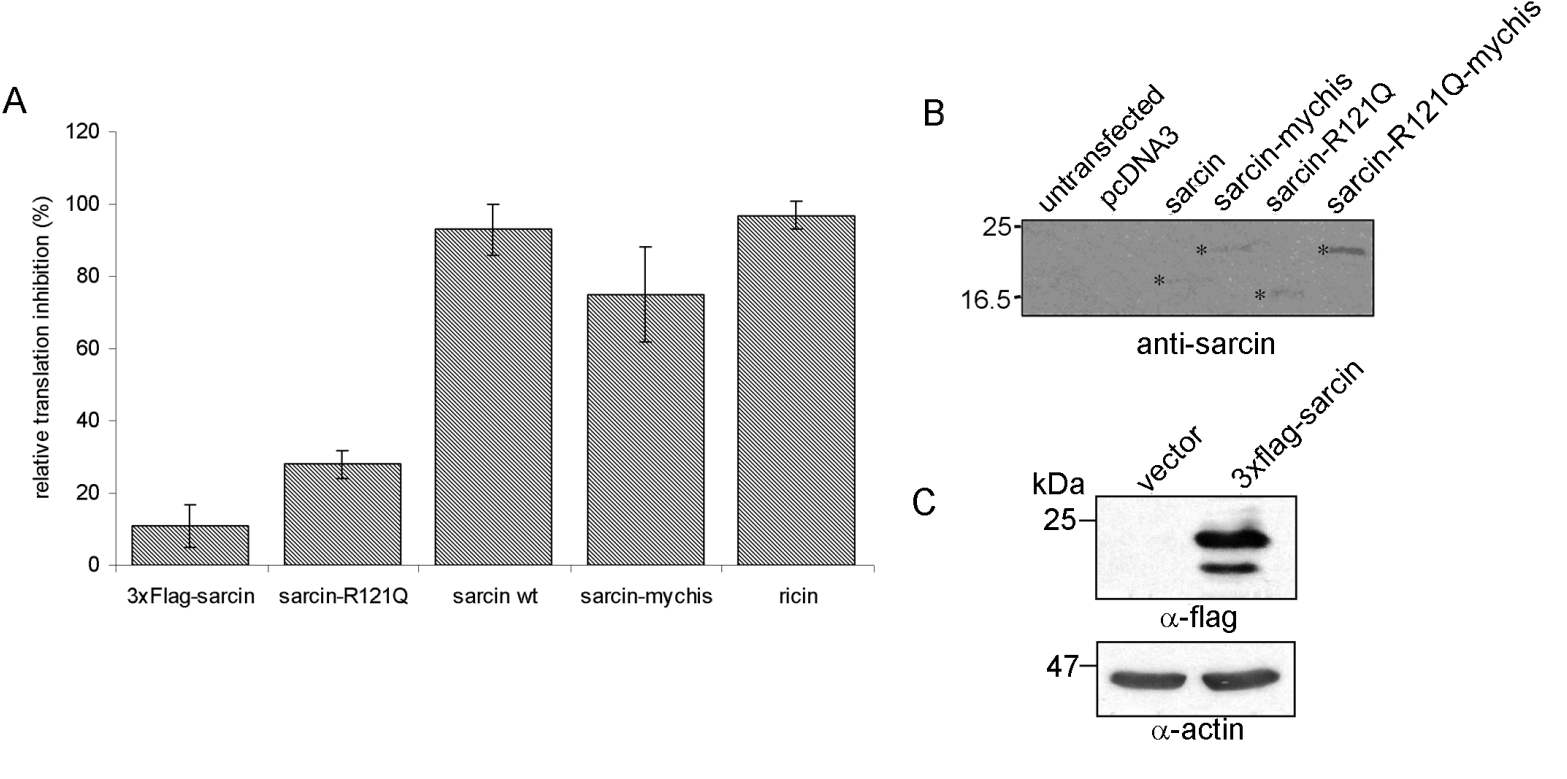
**A) Inhibition of GFP reporter translation mediated by expression of protein toxins**. HeLa cells were transiently co-transfected with indicated toxin cDNAs and a GFP reporter. Inhibition of GFP translation, relative to pcDNA3/GFP co-transfected cells, as determined by fluorimetric analysis of cell lysates. B) Exogenous expression of α-sarcin, α-sarcin-mychis, α-sarcin-R121Q, and α-sarcin-R121Q-mychis in HeLa cells. Anti-sarcin western blot analysis of HeLa cell lysates transfected pcDNA3 or α-sarcin cDNA. * indicates the position of α-sarcin. R121Q mutant migrates slightly faster than wildtype α-sarcin. C) HeLa cells were transfected with either empty vector (MSCV) or 3 × Flag-sarcin. Stable cell lines expressing Flag-tagged-sarcin were established. Anti-Flag western blot of MSCV or 3 × Flag-sarcin transfected cells. Charted data represents the compiled results of 3 independent experiments, each performed in triplicate, +/- standard deviation.

The cytotoxicity of α-sarcin-R121Q suggests that either arginine-121 is not required for protein synthesis inhibition in cells, or that catalytic cleavage of the ribosomal RNA is not necessary for α-sarcin-induced cytotoxicity. To address this issue, we measured the ability of α-sarcin and α-sarcin-R121Q to inhibit the translation of a co-transfected GFP reporter. HeLa cells were transfected with toxin cDNA, as well as a GFP reporter, and the extent of GFP expression was monitored. The transfections were carried out at high cell densities, as this was observed to delay α-sarcin cytotoxicity (data not shown), and therefore, any decrease in GFP levels could be attributed to inhibition of translation, rather than a loss in total number of cells. In addition, cells were examined microscopically prior to analysis to confirm that the monolayers were of similar density, and assays were conducted 18 hours after transfection, before the onset of cell death (Figure 1b). Measurement of GFP fluorescence in cell lysates showed that GFP expression was inhibited relative to a vector control in the wildtype α-sarcin and ricin transfected cells (Figure 2a). Measurement of GFP fluorescence in α-sarcin-R121Q transfected cells revealed that the mutant was attenuated with respect to its ability to inhibit GFP expression, which is consistent with data implicating R121Q involvement in catalysis (Figure 2a) [8]. We also generated an N-terminal 3 × Flag-tagged-α-sarcin (3 × Flag-sarcin) and C-terminal mychis-tagged-α-sarcin (sarcin-mychis). While sarcin-mychis exhibited near wild-type inhibition of GFP translation and toxicity, the 3 × Flag-sarcin was severely attenuated in both translation inhibition and toxicity (Figure 1c, 2a), despite robust expression levels (Figure 2c). The N-terminal 22 amino acids of α-sarcin form a β-hairpin that has been implicated in both the interaction with membranes and in guiding the toxin to the sarcin-ricin loop [12]. The attenuation of toxicity observed with the 3 × Flag-sarcin supports the importance of this region in α-sarcin function.

The finding that α-sarcin-R121Q retained toxicity is contrary to the widely held view that ribotoxins induce apoptosis by blocking protein synthesis [1]. A possible explanation for this result is that the R121Q mutant is expressed to a greater extent. To rule out this possibility, western blot analysis was performed to determine the levels of toxin protein (Figure 2b). The levels of α-sarcin detected using western blotting are invariably low and difficult to detect. Although the levels of the R121Q mutant (both mychis and untagged) were slightly elevated compared to wildtype, α-sarcin-R121Q was expressed at equivalent levels to sarcin-mychis, which was comparable to the wildtype α-sarcin in toxicity and translation inhibition. Therefore, we conclude that the levels of translated toxin are unlikely to account for the observed toxicity of the α-sarcin-R121Q mutant.

**Figure 3:**
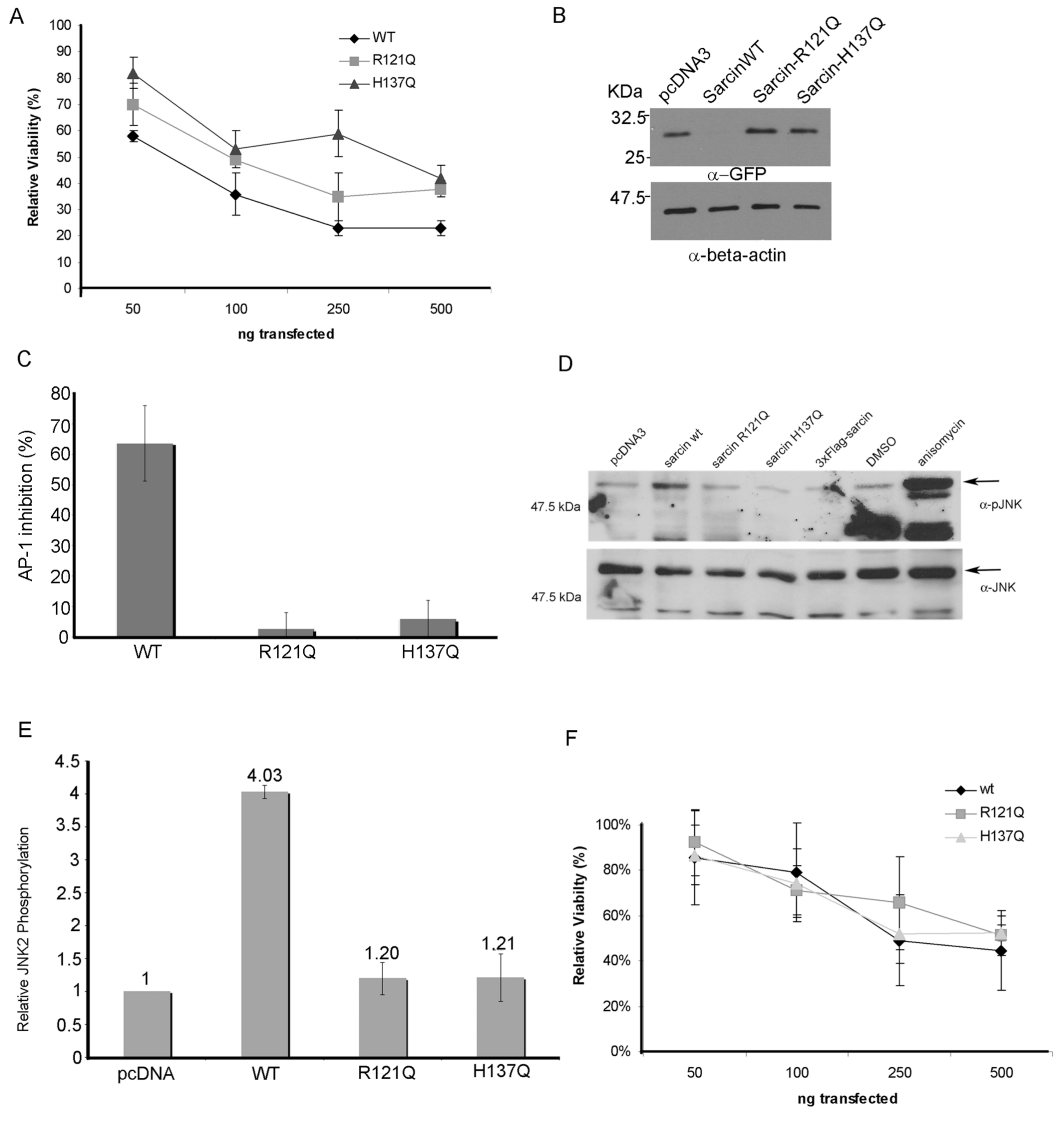
**A) Wild type α-sarcin (wt) and the R121Q and H137Q mutants induced cytotoxicity in HeLa cells in a dose-dependent manner**. HeLa cells were transfected with increasing amounts of toxin cDNA and assayed for viability, relative to a pcDNA3 control, at 48 hours post-transfection. B) Inhibition of GFP translation by α-sarcin wt, R121Q, or H137Q mutants determined by western blotting. C) Inhibition of firefly luciferase expression relative to pcDNA3/luciferase co-transfected cells, as determined by luminescence analysis of cell lysates. D) α-Sarcin wt induces JNK activation whereas the catalytic mutants do not. pJNK Western blot (arrow indicates position of JNK-2) HeLa cell lysates transfected with α-sarcin wt, R121Q mutant, H137Q mutant, 3 × Flag-sarcin or treated with vehicle (DMSO) or anisomycin for 20 minutes. Anisomycin is a potent agonist of JNK and is included as a positive control. (Bottom) For a loading control the above blot was stripped and reprobed with anti-JNK antibody. E) Quantification of JNK activation relative to pcDNA transfected controls as determined using a Li-Cor infrared imager. Charted data represents the compiled results of 3 independent experiments, each performed in triplicate, +/- standard deviation. F) Relative viability of Cos 7 cells transfected with pcDNA3-α-sarcin (wt) or R127Q, or H137Q mutants.

A second possible explanation for the toxicity associated with α-sarcin-R121Q is that only a small decrease in protein synthesis is sufficient to induce cell death in HeLa cells. Therefore to rule out any contribution of residual catalytic activity of the R121Q mutant to the observed toxicity, we tested a second mutant, H137Q. As discussed, Histidine-137 is essential for catalytic activity of α-sarcin, acting as a general acid in the initial transphosphorylation step of the hydrolysis reaction [7]. The H137Q has been shown to be catalytically inactive against ribosomes and dinucleotide substrates in vitro [7]. We tested the H137Q mutant to determine if this catalytically defective mutant was cytotoxic. Direct expression of α-sarcin-H137Q in HeLa cells revealed that, similar to α-sarcin-R121Q, this mutant is cytotoxic (Figure 3a). We measured its ability to inhibit translation of the GFP reporter (Figure 3b) and a second luciferase based reporter, in which luciferase expression is under control of AP-1 promoter. Both GFP and luciferase expression are inhibited by wildtype α-sarcin, but not by either the R121Q or H137Q mutant forms of α-sarcin (Figure 3c). To ensure the cytotoxicity we observed for the α-sarcin-R121Q and H137Q was not restricted to HeLa cells, we also expressed these mutants in Cos7 cells. Direct expression of α-sarcin and the catalytic mutants in Cos7 cells resulted in cytotoxicity (Figure 3F).

Our results are in contrast with a previous study using the H137Q mutant that concluded that α-sarcin toxicity was dependent upon its catalytic activity [5]. An obvious difference between this study and our own is that the previous study relied on adding purified α-sarcin to the media of rhabdomyosarcoma cells and measuring the effect on cell survival and protein synthesis. Since the mechanism of α-sarcin entry and trafficking has not been fully elucidated, one cannot be certain that this mutation does not affect trafficking and, therefore, may not reach the cytoplasm to interact with the ribosome. By using direct cytoplasmic expression, we assessed toxicity independent of trafficking. A second difference between this study and our own is the cell line used. We have chosen two very different cell lines for our study – a human cervical cancer epithelial cell line (HeLa) and an African green monkey fibroblast SV40 transformed cell line (Cos7). Therefore, our data is not cell type specific and the observed differences in cytotoxicity of the α-sarcin mutant between these studies is likely due to a failure of the R121Q and H137Q to properly traffic to the cytoplasm when added to the exterior of the cell.

The 28S rRNA has been proposed to act as a sensor for ribotoxic stress; α-sarcin has been shown to induce Jun N-terminal kinase (JNK) activation and phosphorylation through damage to the sarcin-ricin loop of the 28S rRNA [6]. To test whether this pathway is important for the cytotoxic activity of the mutants, we assayed whether the α-sarcin-R121Q and H137Q mutants could induce JNK activation by measuring JNK phosphorylation. As expected, wild-type α-sarcin induced JNK phosphorylation (Figure 3d, e). However, despite the observed cytotoxicity of the mutants, we did not detect an appreciable activation of this pathway with either catalytic mutant (Figure 3d, e). This is consistent with the notion that 28S rRNA damage is responsible for initiating this stress response [6] and rules out the possible explanation that the mutants retain sufficient activity against the sarcin-ricin loop to induce apoptosis through a JNK mediated stress response. Our results indicate that the cytotoxicity of the mutants is not due to a stress response caused by ribosome disruption.

**Figure 4:**
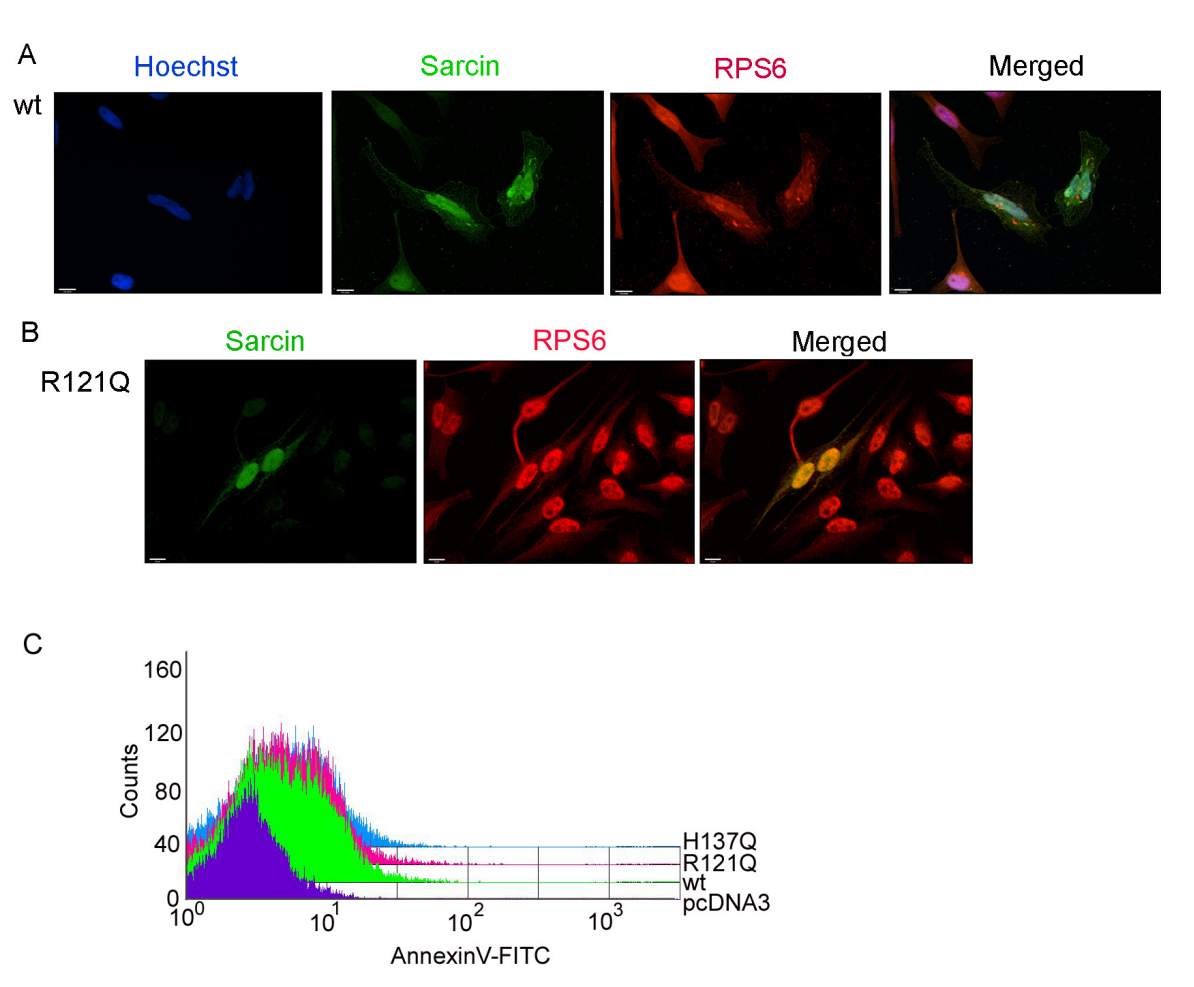
**A) Colocalization of α-sarcin with ribosomal marker RPS6**. HeLa cells were transfected with α-sarcin-mychis cDNA (wt) and after 24 hours, fixed and labeled with anti-myc (FITC channel) and anti-RPS6 (rhodamine channel). (Bar 10 μM) B) Localization of the R121Q-mychis tagged mutant form of α-sarcin. The localization of both mutants appeared identical to wild-type α-sarcin. (Bar 10 μM) C) α-sarcin, and the R121Q and H137Q mutants induce apoptosis. Cos7 cells were transfected with pcDNA3, α-sarcin wildtype (wt), α-sarcin R121Q, or α-sarcin H137Q. After 24 hrs cells were collected and analyzed for annexinV-FITC and propidium iodide staining using FACS.

Recent studies have shown that α-sarcin binds to the ribosome through electrostatic interactions that lie outside of the catalytic site [13,14]. Therefore it is likely the mutants still bind to the ribosome. To test this possibility, we examined the subcellular localization of α-sarcin and the mutants (R121Q shown) in relation to ribosomal marker, RPS6. Both the wild-type α-sarcin and the mutants localized to the nucleus and to a reticulate structure within the cytoplasm, which we determined colocalizes with ribosomal marker RPS6 (Figure 4a, b). A substantial portion of α-sarcin is found within the nucleus. At 17kDa, α-sarcin is expected to diffuse freely through nuclear pore complexes. However, as far as we are aware, this is the first demonstration that α-sarcin accumulates in the nucleus, whether nuclear localization is important for α-sarcin function is an open question.

α-Sarcin has been shown to kill cells by apoptotic induction [5]. To determine whether the cell death we observed by direct expression was due to apoptosis, we measured apoptosis in cells transfected with α-sarcin or each of the mutants. As expected, wild type α-sarcin and both of the mutants induced apoptosis 24 hours after transfection (Figure 4c). We did not detect any difference in necrosis between pcDNA3 controls and wild-type α-sarcin or either of the mutants (data not shown).

We have shown that two independent mutations within the catalytic site of α-sarcin, which impair the catalytic activity of the toxin, do not abrogate α-sarcin cytotoxicity when expressed in cells. We conclude that α-sarcin catalytic activity is not required for α-sarcin cytotoxicity, but it is required to activate JNK in response to ribotoxic stress. This is consistent with reports on the cytotoxicity of N-glycosidase ribosome inactivating proteins, abrin and ricin [15,16]. The N-glycosydic depurination activity of these toxins occurs at a site near to the α-sarcin RNA cleavage site. Abrin has been shown to interact with and inhibit anti-oxidant protein-1 (AOP-1) [15]. This interaction induces apoptosis by triggering an increase in reactive oxygen species, independent of ribosome inactivation. Similarly, in a yeast expression screen, Li et al. identified mutants of ricin, which although catalytically active, were not cytotoxic to yeast cells [16]. This implies that these mutations affect protein interactions other than ricin's catalytic interaction with the ribosome. Whether α-sarcin, which is a ribonuclease, induces apoptosis through interactions with other proteins remains an open question. Since Arginine-121 and Histidine-137 lie within the catalytic site and do not affect electrostatic interactions outside of the active site that are important for ribosomal binding, an alternative explanation for our results is that α-sarcin binding to the ribosome may be more important than the catalytic inactivation of the ribosome [13,14]. The observed co-localization of α-sarcin and mutants with ribosomal markers is consistent with this hypothesis.

It is intriguing that the 3 × Flag epitope on the N-terminus of α-sarcin produced a catalytically inactive innocuous toxin. Furthermore, we found that expression of an α-sarcin with a deleted N-terminus was non-toxic (data not shown). These results implicate the N-terminal β-hairpin loop of α-sarcin in promoting cytotoxicity. Consistent with this hypothesis, Garcia-Ortega et al. have shown that deletion of the entire β-hairpin produces a catalytically active toxin that is non-toxic [12]. This group showed that the N-terminal β-hairpin was important for conferring specificity towards the sarcin-ricin loop and suggested this region may be important for ribosomal recognition. Others have identified a sequence (^15^TNKYETK^21^) within the N-terminus of α-sarcin that is conserved within all members of the ribotoxin family. This resembles a sequence found in elongation factors[17], which is further suggestive that this region may be important in targeting α-sarcin to the ribosome. It is possible that the addition of the 3 × Flag epitope interferes with the ability of the N-terminal β-hairpin to guide the toxin to the sarcin-ricin loop. This would support the idea that binding to the ribosome maybe more important for cytotoxicity than specific cleavage of the sarcin-ricin loop. Alternatively, similar to abrin, α-sarcin may induce apoptosis through direct interaction with a protein (or proteins) in the apoptotic pathway and that the N-terminus of the toxin maybe important for this interaction. Our data cannot exclude either possibility. Intriguing, the N-terminus alone is not sufficient for cytoxicity (data not shown) which suggests that if this region is responsible for cytoxicity, it only functions within the context of the whole protein. Further work is needed to determine how the N-terminus influences cytotoxicity, and whether this is due to interaction with the ribosome or other protein(s). The large-scale mutagenesis approach used by Li et al. to isolate catalytically active, non-toxic ricin mutants based on their inability to kill yeast cells, would be ideally suited for identifying residues that are required for α-sarcin cytotoxicity but not for cleavage of the sarcin-ricin loop [16].

## Conclusion

In conclusion, our results highlight the importance of direct expression analysis when characterizing protein toxin mutations. They indicate that the ribotoxin α-sarcin, similar to the plant ribosome inactivating proteins, ricin and abrin, can directly induce cell death, independent of its ability to inhibit protein synthesis. Thus, our results suggest that α-sarcin can promote cell death through a previously unappreciated mechanism that is independent of rRNA cleavage and JNK activation. This is the first demonstration of this independence within the ribonuclease family. Our results suggest that contrary to the widely held view, protein synthesis inhibition by α-sarcin is insufficient to induce apoptosis and will interest those individuals developing inhibitors of ribotoxins, using ribotoxins for cancer therapy, as well as those interested in understanding ribosome function.

## Methods

### Cloning of α-sarcin and α-sarcin mutants

α-Sarcin cDNA was cloned by consecutive annealing of overlapping primers followed by ligation into *XhoI*- and *HindIII*-sites in pcDNA3.1 or pcDNA3-mychis vector (C-terminal MycHis tag). In addition, α-sarcin cDNA was cloned into 3 × FLAG pMSCV to obtain a N-terminal 3 × Flag tagged version of α-sarcin. α-Sarcin mutants were generated by overlap extension PCR. All α-sarcin clones were characterized by DNA sequencing.

### Cloning of ricin

A *Ricinus communis *specimen bearing seedpods was obtained from a local nursery. Seed coats were removed and approximately 0.5 grams of seed tissue was ground using a small mortar and pestle. The sample was homogenized in TRIzol^® ^reagent (Invitrogen) using a PowerGen 125 homogenizer. TRIzol^® ^extraction of total RNA was performed using an RNA extraction kit according to the manufacturer's protocol (Invitrogen).

Reverse-transcriptase (RT)-PCR reactions were performed according to the manufacturer's instructions (Protoscript^® ^II RT-PCR Kit, New England Biolabs). First strand cDNA synthesis reactions contained nuclease-free water, 100 pmol oligo-dT primer, 200 μM dNTPs, 1× RT buffer, 10 U RNase inhibitor, 750 ng *R. communis *RNA, and 5.0 U M-MuLV Reverse Transcriptase (RT). PCR amplification was performed on 2 μl of cDNA using Deep Vent_R_^TM ^DNA polymerase (New England Biolabs). The ricin cDNA was cloned into *XhoI*- and *HindIII*-treated pcDNA3.1 and sequenced.

### SDS-page and western blotting

Whole cell lysates were harvested, cells were washed once with phosphate-buffered saline (PBS; 0.2 M NaCl, 4.2 mM KCl, 12.7 mM Na_2_HPO_4_, 2.3 mM KH_2_PO_4_) and cell lysis was performed using NP-40 lysis buffer (20 mM Tris pH 8.0, 137 mM NaCl, 10% glycerol, 1% nonidet-P40, 2 mM EDTA).

Whole cell lysates were combined with an equal volume of 2× SDS-PAGE sample buffer (125 mM Tris pH 6.8, 4% SDS, 150 mM DTT, 14% glycerol, 0.04% bromophenol blue) and heated at 95°C for ten minutes prior to electrophoresis. SDS-PAGE was performed according to the method of Laemmli [18]. Protein samples were resolved through 11% SDS-PAGE gels and transferred to nitrocellulose membranes (0.45 μm) using Towbin transfer buffer (25 mM Tris pH 8.3, 192 mM glycine, 20% (w/v) methanol). Membranes were blocked using 5% skim milk in Tris buffered saline (20 mM Tris-HCl pH 7.4, 137 mM NaCl, 0.1% Tween-20). After incubation with primary antibodies, proteins were detected using an Enhanced Chemiluminescence western blotting detection system (Amersham).

### Mammalian cell culture

HeLa and Cos 7 cell lines were cultured in Dulbecco's Modified Eagle Medium (DMEM) with 10% fetal bovine serum, 100 μg/mL penicillin and 100 μg/mL streptomycin at 37°C and 5% CO_2 _according to standard procedures. To assay relative cell viability over time, HeLa cells were seeded at 2 × 10^4 ^cells/well in a 24-well dish. Twenty-four hours later, transient transfection of the appropriate plasmids was carried out using lipofectamine reagent (Invitrogen) according to the manufacturer's instructions. For experiments in which the amount of α-sarcin and ricin cDNAs were varied, the total amount of plasmid in the transfection was held constant by substituting the appropriate amount of pcDNA3.1 vector.

### Protein synthesis inhibition (GFP reporter)

To assay protein synthesis inhibition, HeLa cells were seeded at 3.0 × 10^4 ^cells/well in a 24-well dish. Twenty-four hours later, transient transfection of a GFP reporter plasmid, AdTrack CMV (250 ng), along with the appropriate α-sarcin cDNA, was carried out as described above. Eighteen hours after transfection, cell lysates were harvested and analyzed by fluorimetric analysis (excitation 485 nm/emission 535 nm) using a Victor_TM_^3^V 1420 Multilabel counter (Perkin Elmer). Percentage protein synthesis inhibition was calculated as 1-(RFU_toxin_/RFU_vector control_). Fluorescence analysis represents data from at least three independent experiments performed in triplicate.

### Cell viability assay

To assay dose-dependent killing, HeLa cells were seeded at 2 × 10^4 ^cells/well in a 24-well dish. Twenty-four hours later, transient transfection of the appropriate plasmids was carried out. Amounts of 50, 100, 250, and 500 ng of toxin plasmid DNA were transfected. Where required, control vehicle plasmid (either pcDNA3.1 or pMSCV) was added to the transfection mix to control for the amount of DNA transfected (500 ng per well). Forty-eight hours following transfection, relative cell viability was assayed by crystal violet staining (described below). Briefly, cells cultured in a 24-well dish were washed once with PBS and fixed with 10% formalin for 10 minutes at room temperature. Cells were washed two times with distilled water (dH_2_O) and incubated with 0.1% crystal violet for 30 minutes at room temperature. To remove excess stain, cells were extensively washed with dH_2_O. Crystal violet was extracted using 10% acetic acid. Extracts were diluted 1:4 and their absorbance was measured at 595 nm using a Victor_TM_^3^V 1420 Multilabel plate reader. Percentage viability was determined as the ratio of the A_595 nm _of toxin-transfected cells to the A_595 nm _of pcDNA3.1-transfected control cells. Data represents at least three independent experiments, each performed in triplicate.

### JNK activation assay

HeLa cells were seeded at 1 × 10^5 ^cells/well in 12-well dish (1 mL). Twenty-four hours later, cells were transiently transfected with 1 ug plasmid DNA and Lipofectamine 2000 reagent as per manufacturer's instructions. After 12 hrs of incubation, cells were lysed in 1% NP-40 lysis buffer. For control wells, cells were treated with DMSO (vehicle) or anisomycin (200 ng/mL) for 20 minutes prior to lysis. Phosphorylated JNK and JNK were detected by Western blotting with rabbit anti-pJNK (T183/Y185) and anti-JNK antibodies (Cell Signaling Technologies) and visualized with HRP conjugated secondary antibodies (Jackson ImmunoResearch). JNK activation was quantified using IRDye700DX conjugated secondary antibodies (Rockland) on a Li-Cor Odyssey Infrared Imager running version 2.1 software. Activation of JNK by WT or α-sarcin mutants was made relative to vector (pcDNA3.1) transfected cells. Error bars represent standard deviation of 3 independent analyses.

### α-Sarcin localization

HeLa cells were seeded at 5 × 10^5 ^cells/well in 6-well dish (1 mL) containing a coverglass slip that had been pre-coated with fibronectin. Twenty-four hours later, cells were transiently transfected with 5 ug plasmid DNA and Lipofectamine 2000 reagent as per manufacturer's instructions. After 24 hours, cells were washed in PBS, fixed in 3.7% formalin/PBS, and permeablized with 0.1% Triton X-100. The cells were blocked in 3% BSA/PBS for 1 hour at room temperature and then incubated with either anti-myc (1:500 dilution) and anti-RPS6 (1:500 dilution) antibodies. To detect primary antibody binding, the cells were then incubated with Alexa-Fluor 488 anti-mouse (Molecular Probes) to detect Sarcin-mychis and Rhodamine anti-rabbit (Molecular Probes) to detect RPS6. Images were captured using a Leica DMIRE2 inverted fluorescent microscope with OpenLab V software. Z stacks were imaged at 0.3 uM intervals and deconvolved using nearest neighbor deconvolution.

### Apoptosis assay

Cos7 cells were grown to 75% confluency and transiently transfected as described above using 10 ug of vector (pcDNA 3.1) or α-sarcin (WT or mutant) plasmid DNA and Lipofectamine 2000 according to the manufacturer's instruction. At 24 hours post-transfection, cells were scraped and pelleted, washed once in PBS, and resuspended in 1× Annexin Binding buffer (FITC Annexin V Apoptosis Detection Kit, BD Pharmigen). Cells were stained for flow cytometry using Annexin V-FITC and Propidium Iodide according to manufacturer's instructions (BD Pharmigen) and sorted on a BD FACSCalibur system (BD Biosciences).

### Luciferase assay

HeLa cells were seeded at 3 × 10^5 ^cells/well in a 6-well dish. Twenty-four hours later, cells were transiently transfected with 6.5 ug vector plasmid DNA (background control) or 3 ug pAP1-Luciferase, 0.5 ug pHRCTK (Renilla) and 3.5 ug vector (pcDNA3.1) or α-sarcin (WT or mutant) plasmid DNA and Lipofectamine 2000 reagent as per manufacturer's instructions. After 18 hrs of incubation, cells were removed from the plate by trypsinization and counted. Approximately 1.5 × 10^5 ^cells were taken and luciferase levels determined using the Dual-Glo Luciferase Assay Kit (Promega) on a Victor^3^V Multilabel plate reader. Samples were analyzed in triplicate. Percentage protein synthesis inhibition was calculated as 1-(RLU_toxin_/RLU_vector control_). Error bars represent standard deviation of 3 independent analyses each performed in triplicate.

## Authors' contributions

SA carried out the cytotoxicity and protein synthesis studies, including the cloning of α-sarcin and ricin. JP and RI conceived of and performed the analysis of JNK activity and AP-1 luciferase inhibition. AC carried out the apoptosis and localization studies. PH conceived of the study, participated in its design and coordination, and drafted the manuscript. All authors have read and approved the final manuscript.
